# ﻿Two new species of the *Clistopyga henryi* species-group (Hymenoptera, Ichneumonidae, Pimplinae) from South America, with a key to species of the group

**DOI:** 10.3897/zookeys.1260.163690

**Published:** 2025-11-13

**Authors:** Diego G. Pádua, Ilari E. Sääksjärvi, Kari M. Kaunisto, Ricardo F. Monteiro

**Affiliations:** 1 Laboratorio de Entomología General y Aplicada, Centro de Investigación de Estudios Avanzados del Maule, Vicerrectoría de Investigación y Postgrado, Universidad Católica del Maule – UCM, Talca, Chile Universidad Católica del Maule – UCM Talca Chile; 2 Biodiversity Unit, Zoological Museum, University of Turku, Turku, Finland University of Turku Turku Finland; 3 Laboratório de Ecologia de Insetos, Depto. de Ecologia, Universidade Federal do Rio de Janeiro – UFRJ, Rio de Janeiro, Brazil Universidade Federal do Rio de Janeiro – UFRJ Rio de Janeiro Brazil

**Keywords:** Biodiversity, Darwin wasps, Ephialtini, parasitoid wasps, rainforests, spiders

## Abstract

The Neotropical *Clistopyga henryi* species-group includes currently four described species. In this study, we describe two new species (*C. peruandina* Sääksjärvi & Pádua, **sp. nov.** and *C. teresopolitana* Pádua, **sp. nov.**) from the Peruvian Andes and Brazilian coastal rainforests. In addition, we provide information on the variation of *C. carinata* Bordera & Palacio and a key to species of the species-group.

## ﻿Introduction

*Clistopyga* Gravenhorst, 1829 is a moderately large Darwin wasp genus with 93 valid species (Bordera et al. 2016, 2019, 2025; Yu et al. 2016; Palacio et al. 2018, 2019; Bordera and Palacio 2019; Higa et al. 2024), the great majority of which are distributed in the Neotropical region, followed by the Nearctic, Palearctic, Oriental, and Afrotropical regions (Bordera et al. 2025).

The biology of the genus remains poorly understood but existing evidence suggests that *Clistopyga* represents an evolutionary transition from idiobiont ectoparasitoid wasps that parasitize the silken cocoons of Lepidoptera (Gauld 1991; Gauld and Dubois 2002) to groups that lay eggs in the silken eggs sacs of spiders (Fritzén and Sääksjärvi 2016; Bordera et al. 2025), and ultimately to more derived species-groups functioning as koinobiont ectoparasitoids of active, mobile spiders (Nielsen 1929; Fitton et al. 1988; Wahl and Gauld 1998).

*Clistopyga* is most closely related to *Zaglyptus*, and together with the *Polysphincta* group they form a unique lineage within Ephialtini, characterized by the use of spiders and their egg sacs as a resource for larval development (Gauld and Dubois 2002). Based on morphological characters, the genus has been divided into six species-groups which are all known to occur in the Neotropical region (Gauld 1991; Bordera et al. 2016, 2019, 2025; Palacio et al. 2018, 2019; Bordera and Palacio 2019).

Gauld (1991) was the first to propose the *C. henryi* species-group to host a single species with a long and straight ovipositor (*C. henryi* Gauld, 1991). Palacio et al. (2019) later revised the species-group, described three new species (*C. carinata* Bordera & Palacio, *C. declinata* Palacio & Bordera, and *C. teresitae* Díaz, Palacio & Bordera) from Brazil, Colombia, and Venezuela and provided an additional diagnostic feature for the species-group.

More recently, Bordera et al. (2025) synonymized the Afrotropical genus *Afroanomalia* Varga, 2018 with *Clistopyga* and, consequently, transferred *A. pseudoclistopyga* Varga, 2018 to *Clistopyga* (*C. pseudoclistopyga* (Varga, 2018)), recognizing it as part of the *C. henryi* species-group. Therefore, the genus-group currently contains five species, which are mainly characterized by occipital carina complete, not raised in a flange-like protuberance; fore leg with tibia swollen in their basal half; ovipositor straight or evenly down-curved at distal 0.4; upper valve with evenly tapered, without fine longitudinal rugulae (Varga 2018; Bordera et al. 2025).

In this study, we describe two new species of *C. henryi* species-group, *C. peruandina* Sääksjärvi & Pádua, sp. nov. and *C. teresopolitana* Pádua, sp. nov.) from the Peruvian Andes and Brazilian coastal rainforests, respectively, as well as provide additional information on the morphological variation of the female *C. carinata* and a key for all the Neotropical and Afrotropical species of the genus.

## ﻿Material and methods

The specimens analyzed in this study are deposited in the Departamento de Ecologia e Biologia Evolutiva, São Carlos, São Paulo, Brazil (**DCBU**), Invertebrate Collection of the Instituto Nacional de Pesquisas da Amazônia, Manaus, Brazil (**INPA**), Zoological Museum of the Universidade de São Paulo, São Paulo, São Paulo state, Brazil (**MZUSP**), Entomological Collection of the Universidade Federal do Espírito Santo, Vitória, Espírito Santo, Brazil (**UFES**), Museo de Historia Natural, Universidad Mayor de San Marcos, Lima, Peru (**UNSM**) and Zoological Museum of the University of Turku, Turku, Finland (**ZMUT**).

The morphological terminology follows Broad et al. (2018) and the style of descriptions follows Palacio et al. (2019). The specimens were examined using a Leica EZ4 stereomicroscope (in INPA) and an Olympus SZX10 stereomicroscope (in ZMUT). Measurements were made through a WF10×/22 focusing eyepiece coupled with a 10 mm/100 division reticle and calibrated with a precision ruler.

Digital images were taken using a Leica DFC450 digital camera attached to a Leica M205C stereomicroscope and combined by using the software Leica Application Suite v. 4.6 or Helicon Focus v. 5.3 Pro (in INPA), and using a Canon DS126461 digital camera attached to an Olympus SZX16 stereomicroscope and combined by using the software Zerene Stacker v. 1.04 (in ZMUT) and measurements were made using the software Leica LAS X. Drawings were digitally vectorized using the software Adobe Illustrator.

In this study, the measurements and proportions between the structures are given as the value of the holotype or paratype [in brackets], followed by the minimum and the maximum number of variations if needed. [Brackets] were also used to add, supplement, or correct information on the specimen labels.

The distribution maps were created using SimpleMappr online software (Shorthouse 2010).

## ﻿Results

### ﻿Key to all species of *C. henryi* species-group

**Table:** 

1	Females	**2**
–	Males (males of *C. declinata* Palacio & Bordera, *C. carinata* Bordera & Palacio, *C. teresitae* Díaz, Palacio & Bordera, and *C. peruandina* sp. nov. are unknown)	**8**
2	Ovipositor evenly down-curved at distal 0.4 (Fig. 5A)	**3**
–	Ovipositor straight (Fig. 5B)	**4**
3	Submetapleural carina incomplete, only present at anterior 0.3–0.5 (Fig. 5C, D)	***C. declinata* Palacio & Bordera, 2019**
–	Submetapleural carina strong and complete (Fig. 5E)	***C. carinata* Bordera & Palacio, 2019**
4	Ovipositor < 2.3× as long as hind tibia	**5**
–	Ovipositor > 2.6× as long as hind tibia	**6**
5	Mesosoma predominantly reddish orange, with whitish markings on pronotal, mesopleural, and propodeal regions, and black areas confined to the anterior propleuron, basal metanotum, and median propodeum (Fig. 2A); clypeus 1.3–1.7× as broad as medially long (Fig. 2B)	***C. teresopolitana* Pádua, sp. nov.**
–	Mesosoma predominantly whitish orange, with whitish markings on pronotal, mesopleural, and propodeal regions, and black areas confined to the anterior propleuron, basal metanotum, and median propodeum (Fig. 1A); clypeus 1.9× as broad as medially long (Fig. 1B)	***C. peruandina* Sääksjärvi & Pádua, sp. nov.**
6	Submetapleural carina absent	***C. pseudoclistopyga* (Varga, 2018)**
–	Submetapleural present, incomplete (only present at anteriorly) (Fig. 5C, D)	**7**
7	Metapleuron and propodeum laterally white; tergites II–VIII predominantly red to reddish brown; distal abscissa of *CU* well pigmented (Fig. 5F); ocular orbits usually widely black posteriorly	***C. teresitae* Díaz, Palacio & Bordera, 2019**
–	Metapleuron and propodeum laterally red; tergites II–VIII predominantly black; distal abscissa of *CU* weakly pigmented (Fig. 5G); ocular orbits entirely white	***C. henryi* Gauld, 1991**
8	Hind wing with distal abscissa of *CU* absent	***C. pseudoclistopyga* (Varga, 2018)**
–	Hind wing with distal abscissa of *CU* well pigmented (Fig. 5F) or pigmented weakly (Fig. 5G)	**8**
9	Hind wing with distal abscissa of *CU* well pigmented (Fig. 5F)	***C. henryi* Gauld, 1991**
–	Hind wing with distal abscissa of *CU* weakly pigmented (Fig. 5G)	***C. teresopolitana* Pádua, sp. nov.**

#### ﻿ Clistopyga peruandina

Taxon classificationAnimaliaHymenopteraIchneumonidae

Sääksjärvi & Pádua sp. nov.

D1D48DCD-3B20-5EC9-992B-A60925FD7B0A

https://zoobank.org/CD2CD063-DC78-4475-8631-0AF1468EDDEE

Figs 1A–F, 6

##### Diagnosis.

This species can be distinguished from all other species of the *C. henryi* species-group by the combination of the following characteristics: 1) ovipositor straight; 2) submetapleural carina incomplete, only present at anterior 0.3; 3) hind wing with distal abscissa of *CU* well pigmented; 4) clypeus 1.9× as broad as medially long; 5) metapleuron whitish orange with ventral part weakly blackish and propodeum centrally blackish, lateral part whitish; 6) tergites I–VI blackish with posterolateral black to blackish spots posterior to transverse white bands and 7) ovipositor 2.4× as long as hind tibia; 8) sheath about 2.2× as long as hind tibia, length of setae on average 1.5× the sheath basal width.

**Figure 1.**
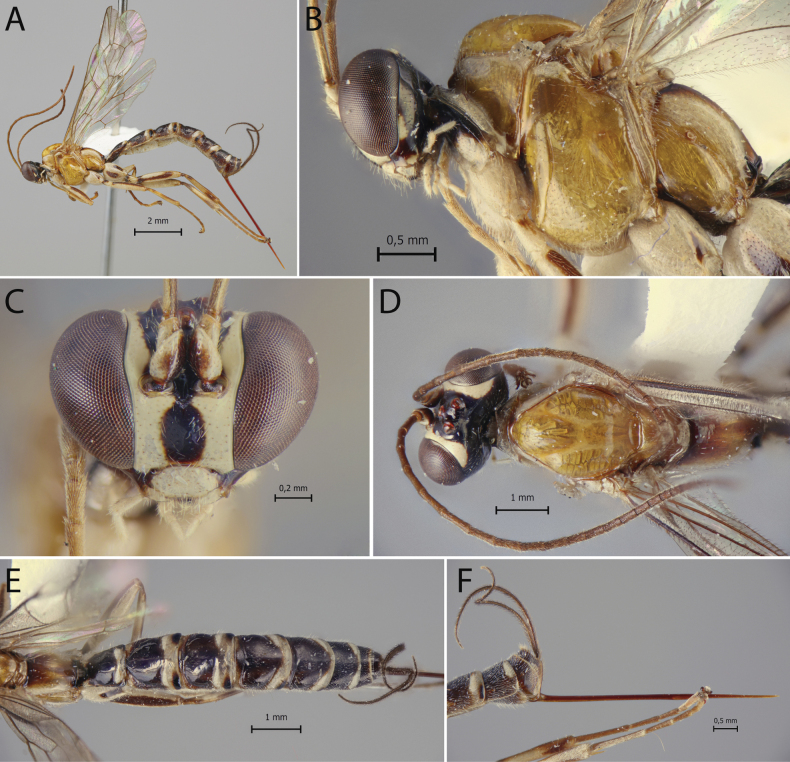
*Clistopyga peruandina* Sääksjärvi & Pádua, sp. nov., female (holotype). A. Habitus; B. Head and mesosoma, lateral view; C. Face, frontal view; D. Head and mesosoma, dorsal view; E. Propodeum and metasoma, dorsal view; F. Ovipositor, lateral view.

##### Description.

**Female**: body length about 10 mm. Fore wing length 6.58 mm. ***Head*.** In dorsal view, strongly narrowed behind the eye. Gena smooth and shiny with evenly sparse setiferous punctures, in dorsal view about 0.6× as long as eye, in frontal view slightly concave and constricted below eye. Frons smooth and shiny. Vertex smooth and shiny, with isolated setiferous punctures. Posterior ocellus separated from eye 0.9× its maximum diameter, the distance between hind ocelli 0.85× its maximum diameter of posterior ocellus. Occipital carina, weak but complete, not raised in a flange-like protuberance at the lower lateral region of the head. Face with fine and relatively scattered setiferous punctures, the distance between punctures is much more than twice the diameter of punctures. Clypeal suture slightly curved. Clypeus 1.9× as broad as medially long, moderately convex in dorsal half, flat in ventral half, with apical margin straight. Malar space about 0.8× as long as basal mandibular width, with a granulate wide sulcus. Antenna with 26 flagellomeres, first flagellomere 5.4× as long as wide. ***Mesosoma*.** Pronotum shiny, smooth, with fine and scattered setiferous punctures except in the center. Mesoscutum entirely smooth and shiny, with fine, sparse setiferous punctures. Notauli deep, reaching 0.4 of length of mesoscutum. Mesopleuron shiny, with very sparse setiferous punctures, except in posterior part below speculum. Epicnemial carina strong, its dorsal end slightly curved backward, ending at the level of centre of pronotum. Metapleuron shiny, almost glabrous, except for some sparse setiferous punctures at posterior part, 1.7× as long as deep. Submetapleural carina incomplete, present at anterior about 0.3. Propodeum smooth and shiny, with sparse and fine setiferous punctures laterally, in dorsal view 1.1× as long as medially wide. Propodeal spiracle joining groove separating propodeum and metapleuron, groove partially interrupted by spiracle. Hind leg with femur 4.6× as long as deep and 0.95× as long as tibia. Fore wing with vein 1*cu-a* more or less antefurcal to *M&Rs*; vein 2*rs-m* about 0.5× as long as abscissa of *M* between 2*rs-m* and 2*m-cu*; the abscissa of *CU* between 1*m-cu&M* and *CU* 1.45× as long as 2*cu-a*. Hind wing with vein *CU* about 0.6× as long as abscissa of *CU* between *M* and *cu-a*; vein *cu-a* reclivous; abscissa of *CU* vertical and straight; distal abscissa of *CU* well pigmented. ***Metasoma*.** Tergite I about 1.1× as long as posteriorly broad, smooth and shiny, with fine, relatively dense setiferous punctures laterally; spiracle near its basal 0.4; lateromedian longitudinal carinae weak, reaching 0.3 of length of tergite; lateral longitudinal carinae weak, reaching 0.3 of length of tergite. Sternite I extending back about 0.6 of length of tergite. Tergite II about 0.9× as long as posteriorly broad, central region shiny, with very fine and very sparse setiferous punctures; anterolateral part weakly rugulose, rest of tergites shiny, progressively more densely and strongly punctate. Ovipositor 2.4× as long as hind tibia, sheath about 2.2× as long as hind tibia, length of setae on average 1.5× the sheath basal width.

**Coloration.** Head black with clypeus, frontal, facial orbits widely and vertical orbits and mouth parts, except apex of mandibles, white; antenna with scape, pedicel black, and first flagelomerus widely white on the outer side, flagellomeres II+ brown, the basal ones pallid on the outer side. Mesosoma mostly whitish orange, upper and lower parts of pronotum, propleuron posteriorly, tegula, subalar prominence, posterior part of mesopleuron, scutellum posteriorly, metanotum dorsally and longitudinal bands laterally of propodeum whitish; propleuron anteriorly, basal part of metanotum and median part of propodeum black. Metasoma mostly black, with transverse white bands on tergites I–VI and whitish areas on anterolateral corners of tergites I–III; tergites II–VI with posterolateral black to blackish spots posterior to transverse white bands. Fore and mid legs predominantly whitish, middle coxae laterally and proximally black, middle trochanters proximally black, femora and tibia dorsally striped with black and tarsi infuscate; hind leg white, coxa laterally and proximally black, trochanter proximally black, femur dorsally and externally striped with black, tibia slightly infuscate, dorsally striped with black, tarsi infuscate. Wings hyaline, pterostigma brown.

**Male.** Unknown.

##### Type material.

***Holotype*: Peru** • ♀, CU [= Cuzco], Cosnipata valley, San Pedro, 24.X.2007, 13°03'23"S, 71°32'55"W, 1520 m, Malaise [trap] 11, C. Castillo leg. (UNSM).

##### Etymology.

The specific name (in apposition) “*peruandina*” refers to the tropical Peruvian Andes.

##### Distribution.

Peru (Fig. 6).

##### Remarks.

*Clistopyga peruandina* sp. nov. resembles *C. carinata* Bordera & Palacio, 2019 and *C. declinata* Palacio & Bordera, 2019 mainly by having metasoma mostly black, with transverse white bands on tergites. However, it clearly differs from both species by straight ovipositor (decurved ovipositor in *C. carinata* and *C. declinata*).

#### ﻿ Clistopyga teresopolitana

Taxon classificationAnimaliaHymenopteraIchneumonidae

Pádua sp. nov.

EC2F897F-051F-5C5A-9F64-8DF5B8995A36

https://zoobank.org/D1AD00C4-74DF-45CD-8452-0DEDEB28CC3D

Figs 2A–F, 6

##### Diagnosis.

This species can be distinguished from all other species of the *C. henryi* species-group by the combination of the following characteristics: 1) ovipositor straight; 2) submetapleural carina incomplete, only present at anterior 0.2; 3) hind wing with distal abscissa of *CU* well pigmented; 4) clypeus 1.3–1.7× as broad as medially long; 5) metapleuron reddish orange with ventral part blackish and propodeum blackish in the center, lateral part whitish; 6) tergites II–VI with posterolateral black to blackish spots posterior to transverse white bands.; 7) female with ovipositor 2.2× as long as hind tibia; 8) sheath about 2.0× as long as hind tibia, length of setae on average 2.0× the sheath basal width.

**Figure 2.**
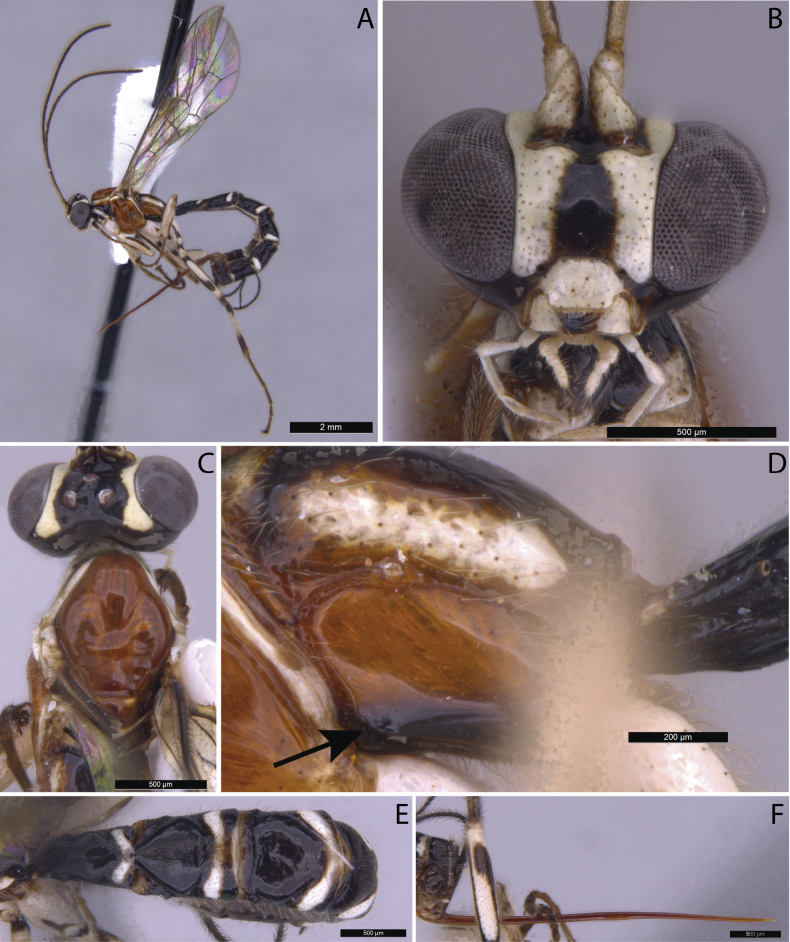
*Clistopyga teresopolitana* Pádua, sp. nov., female (holotype). A. Habitus. B. Face, frontal view; C. Head and mesoscutum, dorsal view; D. Propodeum, lateral view (arrow showing submetapleural carina); E. Metasoma, dorsal view; F. Ovipositor, lateral view.

##### Description.

**Female**: Body length [7.12] 7.0–10.6 mm. Fore wing length [5.12] 5.0–7.15 mm. ***Head*.** In dorsal view, strongly narrowed behind eye. Gena smooth and shiny with evenly sparse setiferous punctures, in dorsal view [0.33] 0.15–0.35× as long as eye, in frontal view slightly concave and constricted below eyes. Frons smooth and shiny. Vertex smooth and shiny, with isolated setiferous punctures. Posterior ocellus separated from eye [0.75] 0.75–1.15× its maximum diameter, distance between hind ocelli [1.33] 1.0–1.35× its maximum diameter of posterior ocellus. Occipital carina, weak but complete, not raised in a flange-like protuberance at the lower lateral region of head. Face with fine and relatively scattered setiferous punctures, distance between punctures much more than twice the diameter of punctures. Clypeal suture slightly curved. Clypeus [1.66] 1.3–1.7× as broad as medially long, moderately convex in dorsal half, flat in ventral half, with apical margin straight. Malar space [0.6] 0.55–0.6× as long as basal mandibular width, with a granulate wide sulcus. Antenna with [25] 25–26 flagellomeres, first flagellomere about [6.0] 6.0–10.5× as long as wide. ***Mesosoma*.** Pronotum shiny, smooth, with fine and scattered setiferous punctures except in the center. Mesoscutum entirely smooth and shiny, with fine and sparse setiferous punctures. Notauli deep, reaching [0.36] 0.25–0.36 of length of mesoscutum. Mesopleuron shiny, with very sparse setiferous punctures, except in posterior part below speculum. Epicnemial carina weak, its dorsal end slightly curved backward, ending at level of centre of pronotum. Metapleuron shiny, almost glabrous, except for some sparse setiferous punctures at posterior part, [1.81] 1.8–1.9× as long as deep. Submetapleural carina incomplete, present at anterior about [0.2]. Propodeum smooth and shiny, with sparse and fine setiferous punctures laterally, in dorsal view [1.2] 1.1–1.2× as long as medially wide. Propodeal spiracle joining groove separating propodeum and metapleuron, groove partially interrupted by spiracle. Hind leg with femur [6.0] 4.0–6.0× as long as deep and [0.88] 0.85–0.9× as long as tibia. Fore wing with vein 1*cu-a* more or less antefurcal to *M&Rs*; vein 2*rs-m* about [0.57] 0.45–0.57× as long as abscissa of *M* between 2*rs-m* and 2*m-cu*; the abscissa of *CU* between 1*m-cu&M* and *CU* [1.14] 1.1–1.5× as long as 2*cu-a*. Hind wing with vein *CU* about [0.5]× as long as abscissa of *CU* between *M* and *cu-a*; vein *cu-a* reclivous; abscissa of *CU* vertical and straight; distal abscissa of *CU* well pigmented. ***Metasoma*.** Tergite I [1.66] 1.4–1.66× as long as posteriorly broad, smooth and shiny, with fine and relatively dense setiferous punctures laterally; spiracle near its basal [0.4]; lateromedian longitudinal carinae weak, reaching about [0.17] 0.15–0.20 of length of tergite; lateral longitudinal carinae weak, reaching about [0.33] of the length of tergite. Sternite I extending back [0.46] 0.4–0.5 of the length of tergite. Tergite II [1.16] 1.1–1.3× as long as posteriorly broad, central region shiny, with very fine and very sparse setiferous punctures; anterolateral part weakly rugulose, rest of tergites shiny, progressively more densely and strongly punctate. Ovipositor [2.2]× as long as hind tibia, sheath about [2.0]× as long as hind tibia, length of setae on average [2.0]× the sheath basal width.

**Coloration.** Head black with, frontal, facial orbits widely and vertical orbits and mouth parts, except apex of mandibles, white; antenna with scape and pedicel black, widely white on the outer side, flagellomeres brown, the basal ones pallid on the outer side. Mesosoma mostly reddish orange, upper and lower parts of pronotum, propleuron posteriorly, tegula, subalar prominence, posterior part of mesopleuron, scutellum posteriorly, metanotum dorsally and longitudinal bands laterally of propodeum whitish; propleuron anteriorly, basal part of metanotum and median part of propodeum black. Metasoma mostly black, with transverse white bands on tergites I–VI and whitish areas on anterolateral corners of tergites I–III; tergites II–VI with posterolateral black to blackish spots posterior to transverse white bands. Fore and mid legs predominantly whitish, middle coxae laterally and proximally black, middle trochanters proximally black, femora and tibia dorsally striped with black and tarsi infuscate; hind leg white, coxa laterally and proximally black, trochanter proximally black, femur dorsally and externally striped with black, tibia slightly infuscate, dorsally striped with black, tarsi infuscate. Wings hyaline, pterostigma brown.

**Figure 3.**
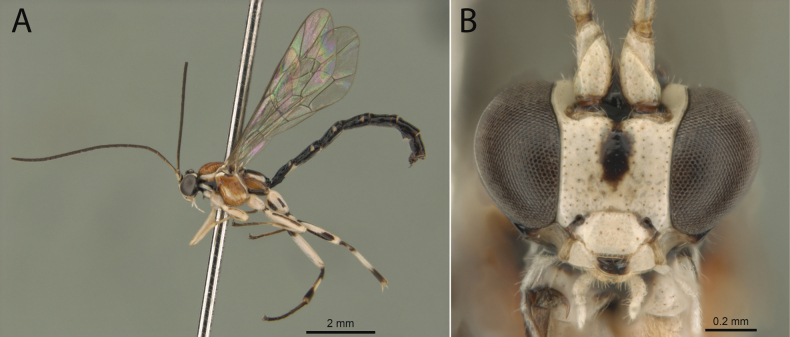
*Clistopyga teresopolitana* Pádua, sp. nov., male (paratype). A. Habitus; B. Face, frontal view.

**Male.** Similar to female in structure and coloration (Fig. 3), except by body length 6.0–8.5 mm; fore wing length 4.0–6.5 mm; ventral part of gena with longitudinal conspicuous concavity; eyes with strong concavity, 0.3–0.4× as long as eye; posterior ocellus separated from the eye about 0.85–1.0× its maximum diameter; distance between hind ocelli about 1.0–1.2× maximum diameter of posterior ocellus; clypeus 0.55–0.6× as broad as medially long, weakly concave ventrally; antenna with 22–24 flagellomeres, first flagellomere. 6.0–7.0× as long as wide; metapleuron 2.0–2.3× as long as deep; hind leg with femur 4.8–6.5× as long as deep; vein 2*rs-m* about 0.4× as long as abscissa of *M* between 2*rs-m* and 2*m-cu*; tergite I and tergite II, 1.75–1.8 and 1.6–1.7× as long as posteriorly broad, respectively; malar space yellowish; metasoma mostly black with tergites I–IV with posterolateral blackish spots.

##### Type material.

***Holotype*: Brazil**, • ♀, RJ [= Rio de Janeiro], Teresópolis, PARNASO [= Parque Nacional da Serra dos Órgãos], Pto. 6B, 877 m., 22°28'11.5"S, 43°00'06.0"W, VI.2015, [Malaise trap] (R.F. Monteiro and team leg.), DCBU. ***Paratypes***: • same data of holotype, but. IX.2015, 1♀, DCBU; • idem, but Pto. 13B, 1941 m., 22°27'17.8"S, 43°01'12.2"W, IX.2015, 1♀, INPA; • idem, but IX.2015, 1♀, DCBU; • idem, but Pto. 15A, 2140 m., 22°27'37.4"S, 43°01'42.9"W, V.2015, 1♂, MZUSP; idem, but • 1♂, DCBU.

##### Etymology.

The specific name “*teresopolitana*” is an adjective referring to the type locality, Teresópolis, Rio de Janeiro, Brazil. The toponym, meaning “city of Teresa,” combines the personal name Teresa (of Greek origin) with the Greek word πόλις (pólis, “city”).

##### Distribution.

Brazil (Fig. 6).

##### Remarks.

*Clistopyga teresopolitana* sp. nov. resembles *C. henryi* Gauld, 1991 mainly by body coloration (reddish-orange in general), submetapleural carina not complete, and ovipositor straight, not decurved. However, it clearly differs from *C. henryi* by its shorter ovipositor (2.2× as long as the hind tibia, ovipositor 2.7–2.8× as long as hind tibia in *C. henryi*).

#### ﻿ Clistopyga carinata

Taxon classificationAnimaliaHymenopteraIchneumonidae

Bordera & Palacio, 2019

B252F7EF-7065-5173-8F7C-068F31BFE62C

Figs 4, 6

Clistopyga carinata Bordera & Palacio, 2019: 105. Holotype: ♀, Brazil (AEIC).

##### Diagnosis.

According to Palacio et al. (2019), this species can be distinguished from all other species of *C. henryi* group by the combination of the following characteristics: 1) hind wing with distal abscissa of *CU* well pigmented; 2) metapleuron and propodeum reddish-orange; 3) submetapleural carina complete; 4) tergites II–VIII predominantly dark brown; 5) female with ovipositor evenly down curved at distal 0.4.

**Figure 4.**
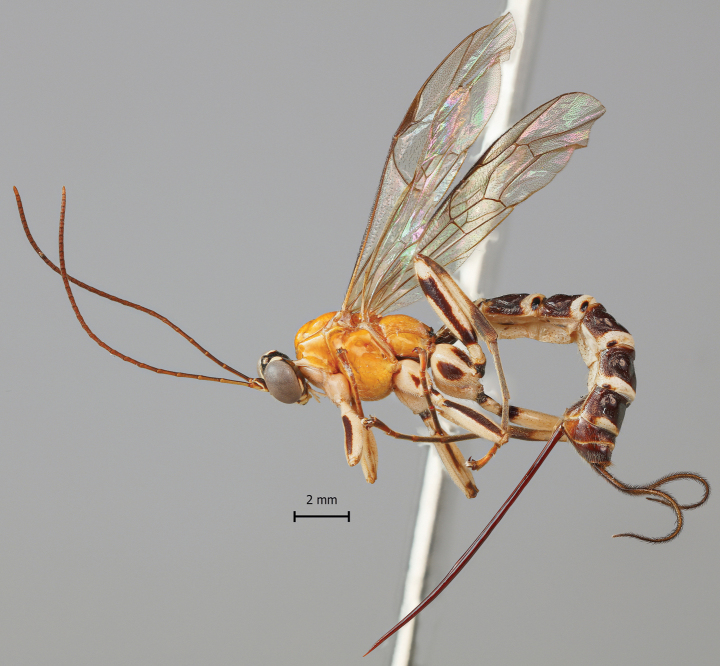
*Clistopyga carinata* Bordera & Palacio, 2019, female, habitus.

**Figure 5.**
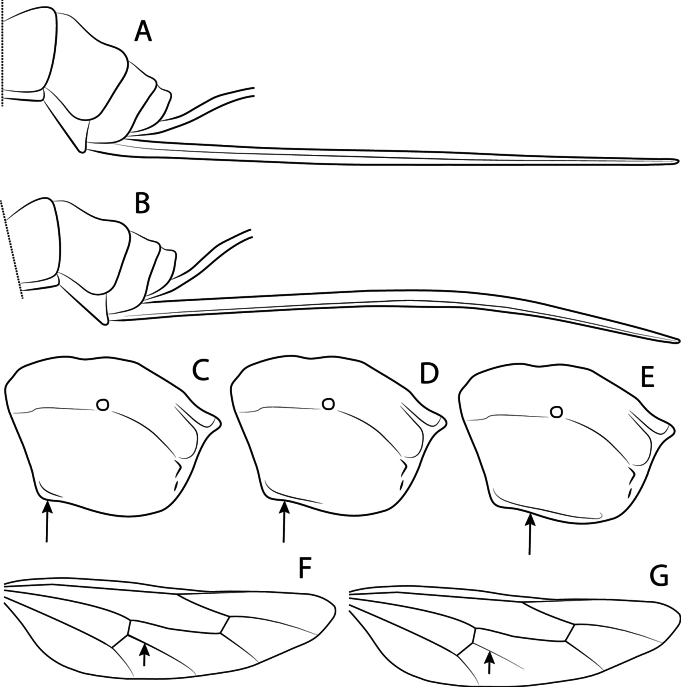
*Clistopyga* spp. morphological structures. A, B. Metasomal apex and ovipositor, lateral view; C–E. Propodeum, lateral view (arrows showing submetapleural carina); F, G. Hind wing, lateral view (arrows showing the distal abscissa of *CU*).

##### Material examined.

**Brazil** • RJ [= Rio de Janeiro], Guapimirim, PARNASO [= Parque Nacional da Serra dos Órgãos], Pto. 4B, 540 m., 22°28'36.5"S, 42°59'30.8"W, XII.2014, [Malaise trap] (R.F. Monteiro and team leg.), 1♀, DCBU; • idem, but Guapimirim, Pto. 3A, 332 m., 22°29'40.4"S, 42°59'52.6"W, VII.2015, 1♀, DCBU; • idem, but IX.2015, 1♀, MZUSP; • idem, but Pto. 4A, 540 m., 22°28'36.5"S, 42°59'30.8"W, 1♀, DCBU; • idem, but V.2015, 1♀, DCBU; • idem, but IX.2015, 1♀, DCBU; • idem, but XI.2015, 1♀, DCBU; • idem, but Teresópolis, Pto. 5A, 877 m., 22°28'37.4"S, 42°59'45.0"W, VIII.2015, 1♀, DCBU; • idem, but Pto 5B, 703 m., 22°28'37.5"S, 42°59'45.1"W, IV.2015, 1♀, MZUSP; • idem, but Pto 7A, 952 m., 22°27'24.8"S, 42°59'07.2"W, 1♀, MZUSP; • idem, but Pto 9A, 1236 m., 22°28'57.8"S, 43°00'13.7"W, III.2015, 1♀, INPA; • idem, but IV.2015, 1♀, INPA; • idem, but Pto 10A, 1444 m., 22°26'51.0"S, 43°00'46.4"W, 1♀, INPA; • idem, but Pto 10B, 1482 m., 22°26'54.2"S, 43°00'49.0"W, IX.2015, 2♀♀, INPA; • idem, but IV.2015, • 1♀, INPA; ES [= Espírito Santo], Dom. [= Domingos] Martins, Mata Pico do Eldorado, 20°22'17"S, 40°39'29"W, 03–10.XII.2004, Malaise [trap] B4 (M.T. Tavares & Eq. col); • det. I. Silva-Santos, 2025, 1♀ #104701, UFES.

##### Distribution.

**Figure 6.**
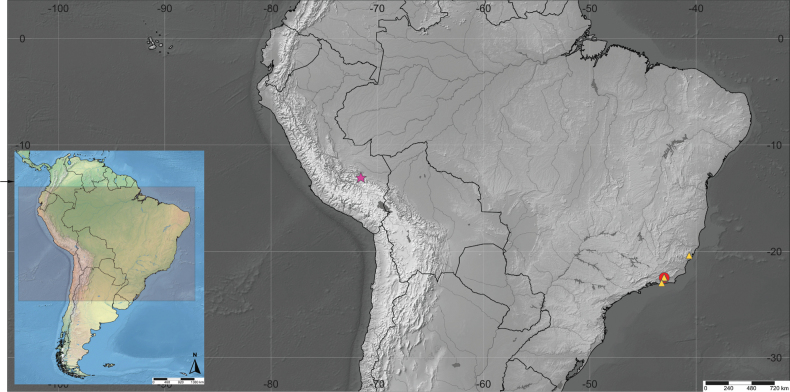
Geographical distribution. Purple star: *Clistopyga peruandina* Sääksjärvi & Pádua, sp. nov.; yellow triangle: *C. carinata* Bordera & Palacio, 2019; red circle: *C. teresopolitana* Pádua, sp. nov.

**Brazil** (Rio de Janeiro and Espírito Santo) (Fig. 6).

##### Remarks.

After studying a large number of specimens, we report the following morphological variations in comparison with the original description by Bordera and Palacio (2019): female. Body length 8.9–12.0 mm. Fore wing length 6.7–8.6 mm. Gena, in dorsal view about 0.18–0.45× as long as the eye. Posterior ocellus separated from the eye 1.0–1.25× its maximum diameter; the distance between the hind ocellus about 1.0–1.18× its maximum diameter of the posterior ocellus. Clypeus 1.6–1.88× as broad as medially long. Malar space about 0.57–0.66× as long as basal mandibular width. Antenna with 26–28 flagellomeres, first flagellomere 7.0–9.0× as long as wide. Notauli reaching 0.35–0.45 of the length of the mesoscutum. Metapleuron 1.66–1.81× as long as deep. Propodeum, in dorsal view 0.92–1.0× as long as medially wide. Hind leg with femur 4.55–5.4× as long as deep and about 0.93–1.0× as long as the tibia. Fore wing with vein 2*rs-m* about 0.33–0.66× as long as abscissa of *M* between 2*rs-m* and 2*m-cu*; abscissa of *CU* between 1*m-cu&M* and *CU* 1.58–1.71× as long as 2*cu-a*. Hind wing with vein *CU* 0.54–0.67× as long as abscissa of *CU* between *M* and *cu-a*. Tergite I 1.0–1.33× as long as posteriorly broad; lateromedian longitudinal carinae reaching 0.2–0.3 of the length of tergite; lateral longitudinal carinae reaching 0.2–0.3 of the length of tergite. Sternite I extending back about 0.4–0.5 of length of tergite. Tergite II 0.93–1.05× as long as posteriorly broad. Ovipositor 2.6–3.0× as long as hind tibia; sheath about 2.05–2.4× as long as the hind tibia, length of setae on average about 2.0–2.5× the sheath basal width.

## Supplementary Material

XML Treatment for Clistopyga peruandina

XML Treatment for Clistopyga teresopolitana

XML Treatment for Clistopyga carinata

## ﻿Additional information

### Use of AI

No use of AI was reported.

### Author ORCIDs

Diego G. Pádua https://orcid.org/0000-0001-5061-2978

Ilari E. Sääksjärvi https://orcid.org/0000-0002-8107-5607

Kari M. Kaunisto https://orcid.org/0000-0001-6665-0047

Ricardo F. Monteiro https://orcid.org/0000-0002-5137-9693

### Data availability

All of the data that support the findings of this study are available in the main text.

## References

[B1] Bordera S Palacio E (2019) The Neotropical species of *Clistopyga* (Hymenoptera, Ichneumonidae, Pimplinae). Part IV: The *C. eldae* species group, with the description of three new species. Zootaxa 4564(2): 327–346. 10.11646/zootaxa.4564.2.2 31716501

[B2] Bordera S Sääksjärvi IE Castillo C Palacio E González-Moreno A (2016) The Neotropical species of *Clistopyga* (Hymenoptera, Ichneumonidae, Pimplinae). Part I. The *C. chaconi* species group, with the description of eleven new species. European Journal of Taxonomy 206(206): 1–37. 10.5852/ejt.2016.206

[B3] Bordera S Palacio E Martínez JJ (2019) The Neotropical species of *Clistopyga* (Hymenoptera, Ichneumonidae, Pimplinae). Part V. The *C. diazi* species group, with the description of three new species. Zootaxa 4661(3): 545–565. 10.11646/zootaxa.4661.3.8 31716702

[B4] Bordera S Palacio E Herrera-Flórez AF (2025) The Neotropical species of *Clistopyga* (Hymenoptera, Ichneumonidae, Pimplinae). Part VI: The *C. calixtoi* species group, with the description of twenty-four new species. Zootaxa 5662(1): 1–115. 10.11646/zootaxa.5662.1.1 41119811

[B5] Broad GR Shaw MR Fitton MG (2018) Ichneumonid Wasps (Hymenoptera: Ichneumonoidea): Their Classification and Biology. RES Handbooks for the Identification of British Insects 7(12). Field Studies Council, Shrewsbury Handbooks Identification British Insects 7(1): 1–110. 10.1079/9781800625471.0000

[B6] Fritzén NR Sääksjärvi IE (2016) Spider silk felting – functional morphology of the ovipositor tip of *Clistopyga* sp. (Ichneumonidae) reveals a novel use of the hymenopteran ovipositor. Biology Letters 12(8): 20160350. 10.1098/rsbl.2016.0350 27512134 PMC5014030

[B7] Gauld ID (1991) The Ichneumonidae of Costa Rica, Volume 1: Keys to subfamilies, and keys to the species of the lower pimpliform subfamilies Rhyssinae, Pimplinae, Poemeniinae, Acaenitinae and Cylloceriinae 1. Memoirs of the American Entomological Institute 47: 1–589.

[B8] Gauld ID Dubois J (2002) Phylogeny of the *Polysphincta* group of genera (Hymenoptera: Ichneumonidae; Pimplinae): a taxonomic revision of spider ectoparasitoids. Systematic Entomology 31(3): 529–564. 10.1111/j.1365-3113.2006.00334.x

[B9] Higa PT Victorino BI Dias MM Penteado-Dias AM (2024) New species and new records of *Clistopyga* (Ichneumonidae: Pimplinae) in Brazil. Zootaxa 5523(3): 354–362. 10.11646/zootaxa.5523.3.3 39645933

[B10] Nielsen E (1929) A second Supplementary Note on the Life Histories of the Polysphinctas. Entomologiske Meddelelser 16: 366–368.

[B11] Palacio E Bordera S Sääksjärvi IE Díaz F (2018) The Neotropical species of *Clistopyga* (Hymenoptera, Ichneumonidae, Pimplinae). Part II: The *C. isayae* species group, with the description of seven new species. Zootaxa 4442(1): 101–121. 10.11646/zootaxa.4442.1.5 30313985

[B12] Palacio E Bordera S Díaz F (2019) The Neotropical species of *Clistopyga* (Hymenoptera, Ichneumonidae, Pimplinae). Part III: The *C. henryi* species group, with the description of three new species. Zootaxa 4564(1): 103–118. 10.11646/zootaxa.4563.1.5 31716501

[B13] Shorthouse DP (2010) SimpleMappr, an online tool to produce publication-quality point maps. http://www.simplemappr.net/ [Accessed 2020 September 09]

[B14] Varga O (2018) *Afroanomalia pseudoclistopyga*, a new genus and species of pimpline parasitoid wasp (Hymenoptera: Ichneumonidae: Pimplinae) from the Afrotropical region. Zootaxa 4461(1): 146–150. 10.11646/zootaxa.4461.1.12 30314104

[B15] Wahl DB Gauld ID (1998) The cladistics and higher classification of the Pimpliformes (Hymenoptera: Ichneumonidae). Systematic Entomology 23(3): 265–298. 10.1046/j.1365-3113.1998.00057.x

[B16] Yu DS van Achterberg C Horstmann K (2016) World Ichneumonoidea 2015: Taxonomy, Biology, Morphology, and Distribution. Taxapad 2016. Database on flash drive.

